# 

**Published:** 1889-08

**Authors:** 


### CONTENTS.

COMMUNICATIONS.

The President's Address.......... 369
Etiology of Caries.......... 390
Reflex Neurosis in Relation to Dental Pathology.......... 398
Dental Trade Association.......... 407

SELECTIONS.

The Alcoholic and Opium Habit.......... 412
A Simple Inhaler.......... 414
Quinine and Tannic Acid.......... 415

EDITORIAL.

An Interesting Case.......... 416
A Suggestive Case.......... 417
Explanatory.......... 419
Painless Destruction of Nævi.......... 420

